# Supplementary figures and images for: Chemokine signaling links cell-cycle progression and cilia formation for left–right symmetry breaking
Source: PLoS Biol. 2019 Aug 20;17(8):e3000203. doi: 10.1371/journal.pbio.3000203 (PMC6716676; doi:10.1371/journal.pbio.3000203)

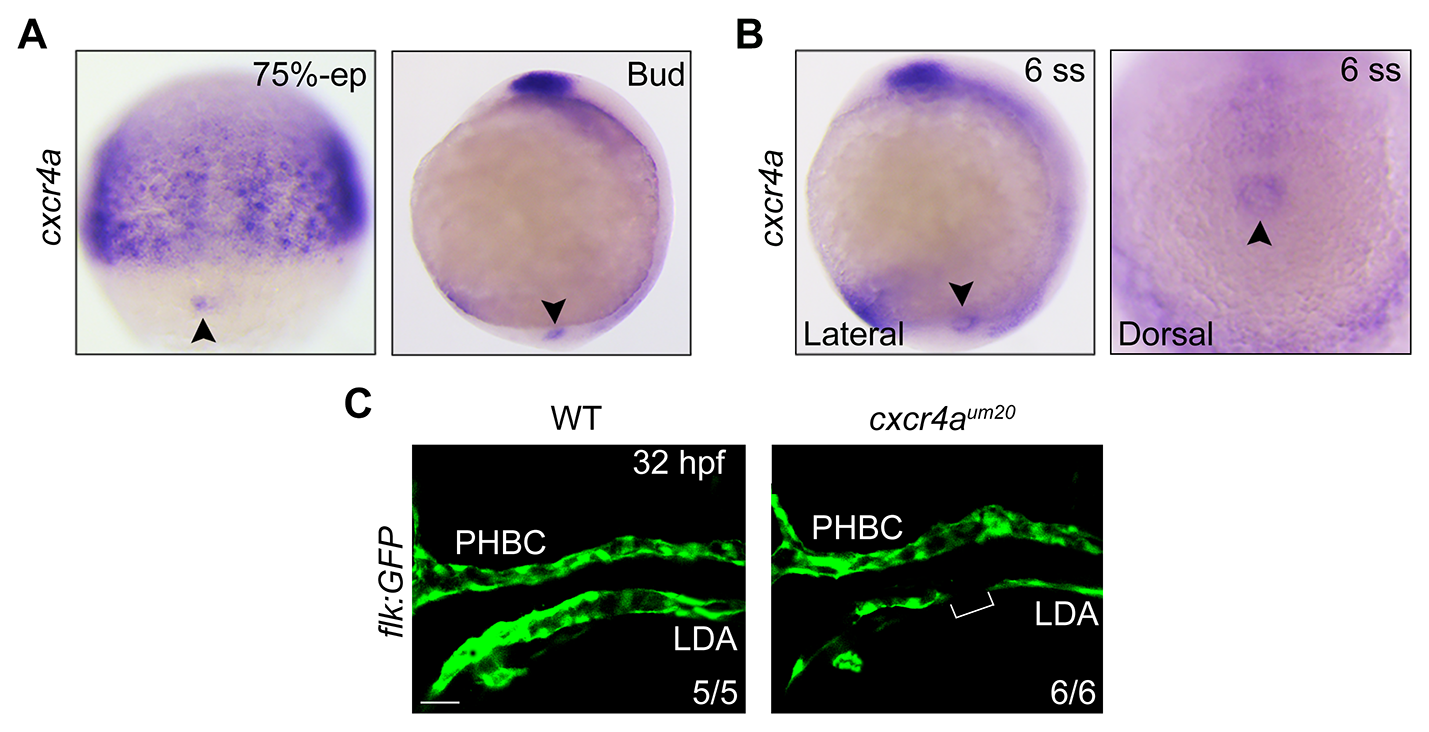

Supplement: S1 Fig — (A) cxcr4a expression during gastrulation. In situ hybridization of cxcr4a in embryos at the 75% epiboly stage (dorsal view with animal pole to the top) and bud stage (lateral views with animal pole to the top). Black arrowhead indicates the DFCs. (B) cxcr4a expression at the 6-somite stage. Lateral view was shown with animal pole to the top in the left panel, and dorsal view was shown in the right panel. Black arrowhead indicates the KV. (C) Confocal images depicting the formation of the lateral dorsal aorta in live Tg(flk:GFP) embryos. Scale bar, 50 μm. DFC, dorsal forerunner cell; ep, epiboly; flk, fms-like tyrosine kinase; GFP, green fluorescent protein; KV, Kupffer’s vesicle; LDA, lateral dorsal aorta; PHBC, primordial hindbrain channel; Tg, transgene. (TIF) [file pbio.3000203.s001.tif]

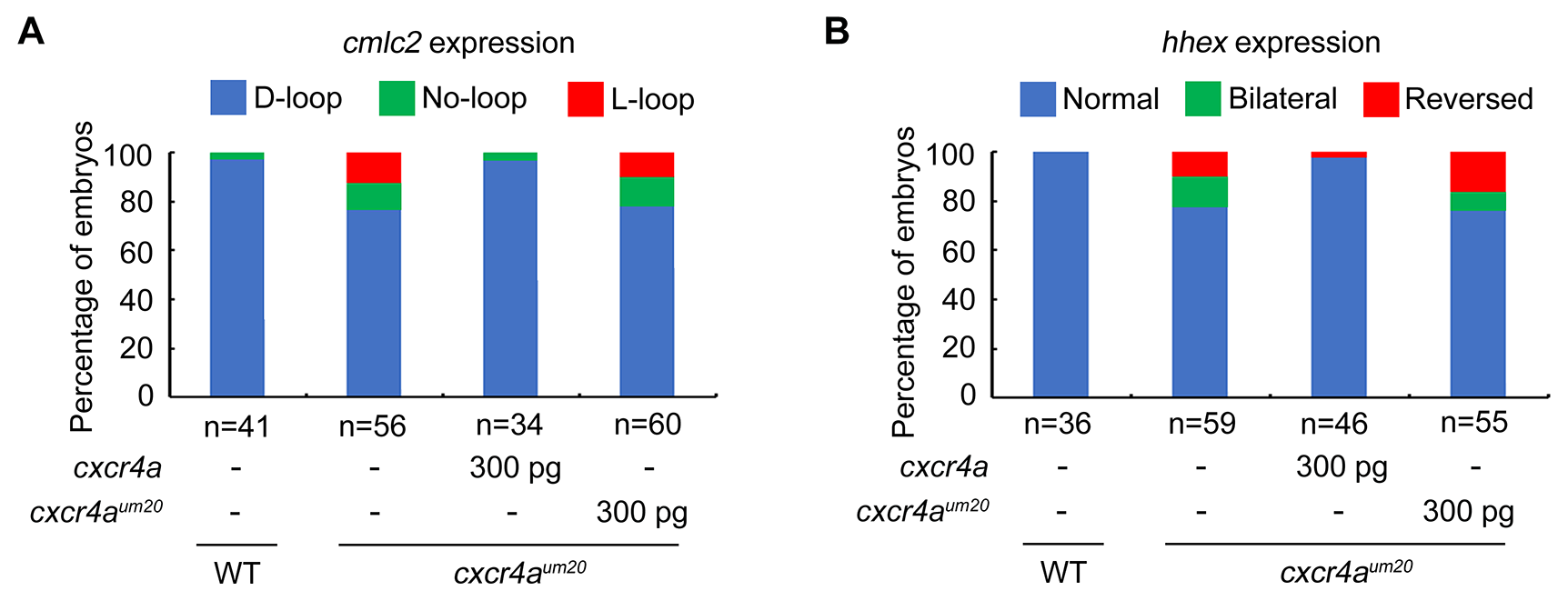

Supplement: S2 Fig — (A–B) Embryo ratios with different expression patterns of cmlc2 (A) and hhex (B) at 48 hpf in cxcr4aum20 mutants injected with 300 pg cxcr4a or cxcr4aum20 mRNA at the 256-cell stage. Underlying data can be found in S1 Data. cmlc2, cardiac myosin light chain 2; hhex, hematopoietically expressed homeobox; hpf, hours postfertilization. (TIF) [file pbio.3000203.s002.tif]

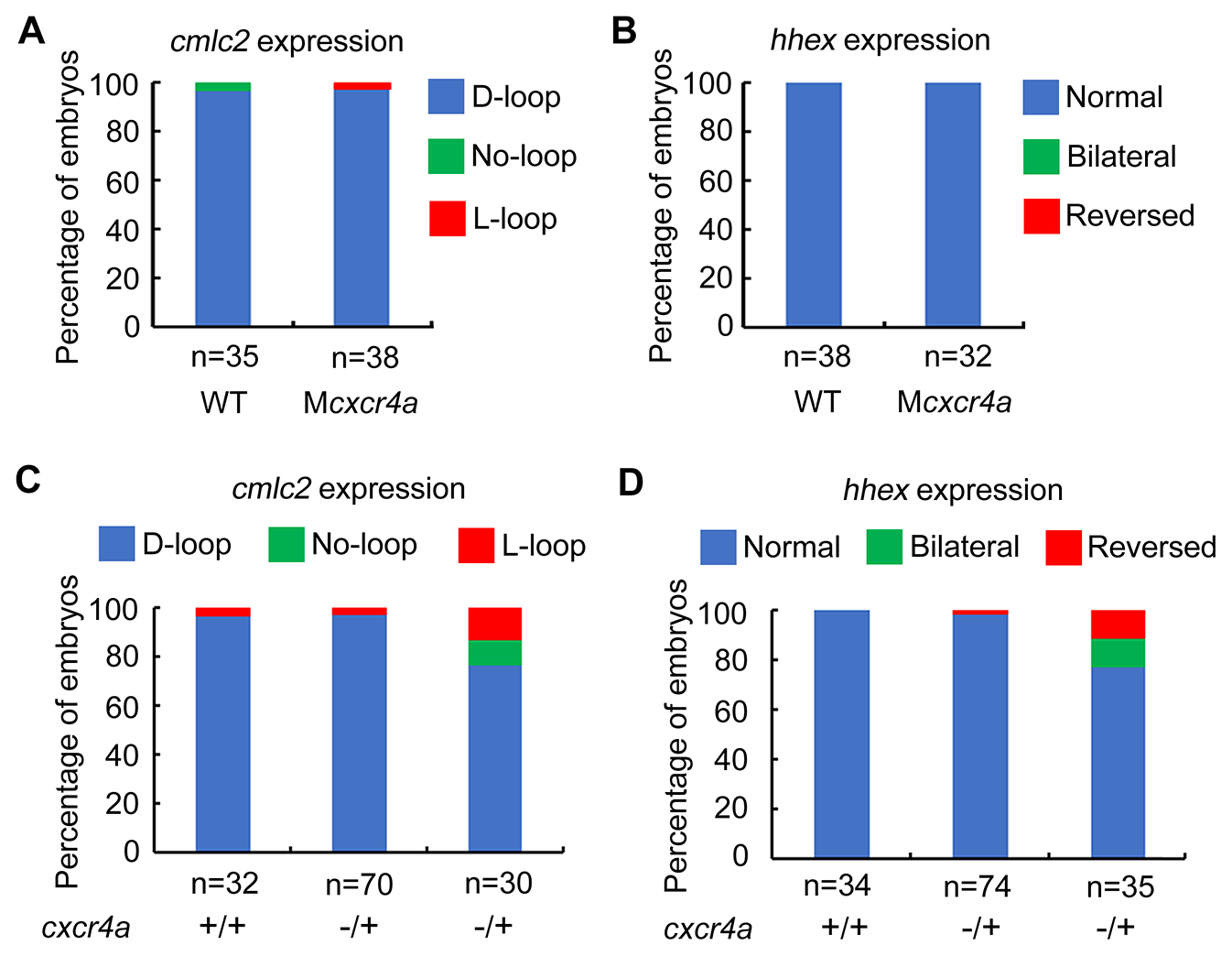

Supplement: S3 Fig — (A–B) The percentage of embryos with different phenotypes in cardiac looping (A) and liver laterality (B). The maternal cxcr4a mutants (Mcxcr4a) were generated by crossing MZcxcr4a mutant adult females with wild-type male fish. Underlying data can be found in S1 Data. (C–D) Analysis of cardiac looping (C) and liver laterality (D) in wild-type (cxcr4a+/+) embryos and zygotic cxcr4aum20 heterozygous (cxcr4a−/+) or homozygous (cxcr4a−/−) mutants. cxcr4a−/+ and cxcr4a−/− embryos were identified from cxcr4aum20 heterozygous fish crosses by genotyping. Underlying data can be found in S1 Data. L–R, left–right. (TIF) [file pbio.3000203.s003.tif]

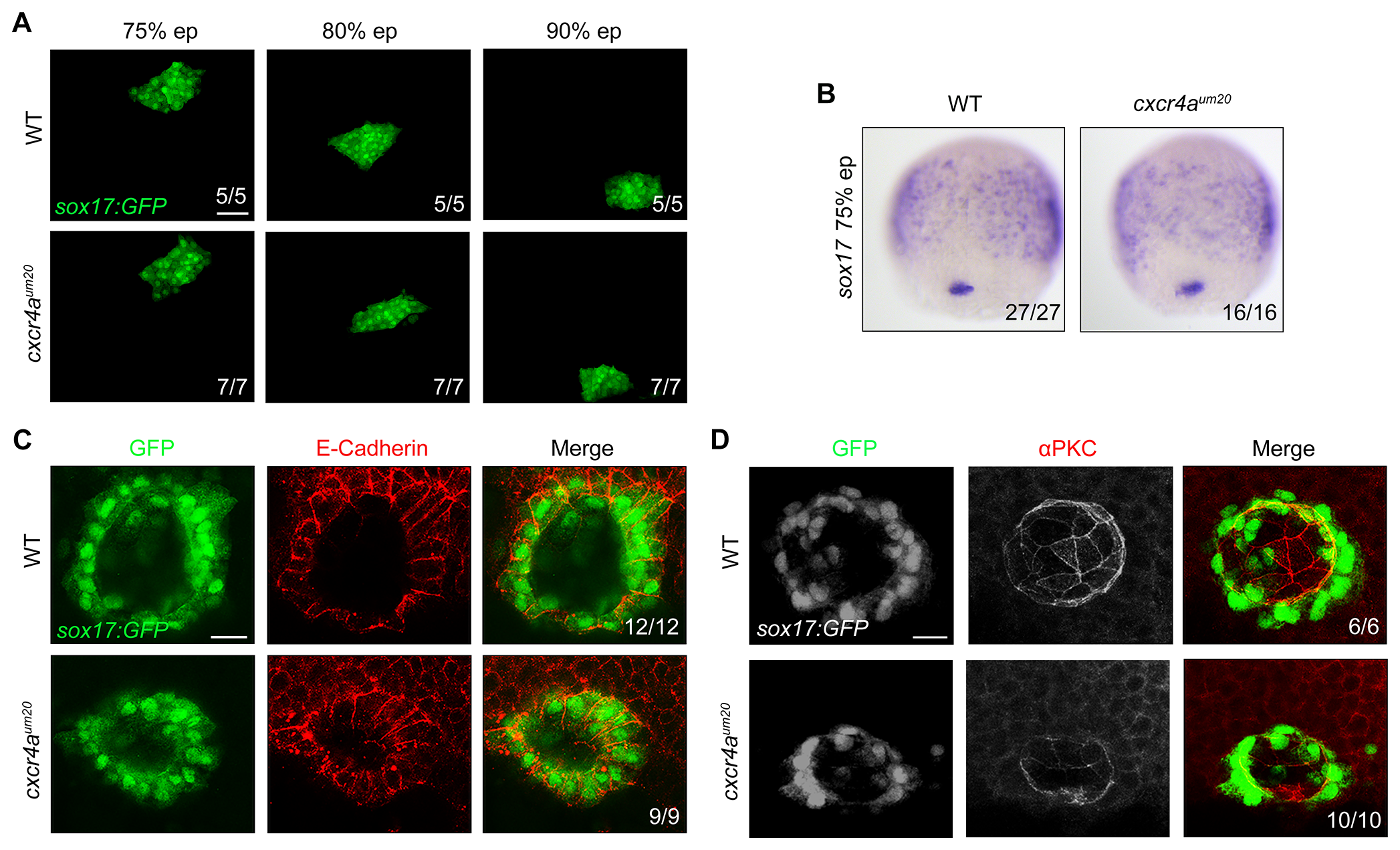

Supplement: S4 Fig — (A) Time-lapse confocal images showing DFC migration in wild-type and cxcr4aum20 mutant embryos on a Tg(sox17:GFP) background from 75%–90% epiboly stages. Scale bar, 50 μm. (B) Sox17 expression was examined by in situ hybridization in wild-type and cxcr4aum20 mutants at the 75% epiboly stage. (C–D) Wild-type and cxcr4a-deficient Tg(sox17:GFP) embryos were harvested at the 10-somite stage for immunostaining. KV cells were labeled using an antibody against GFP. Expression of the basal–lateral marker E-cadherin (C) and the apical marker aPKC (D) were visualized using the indicated antibodies. Scale bar, 20 μm. aPKC, atypical protein kinase; DFC, dorsal forerunner cell; GFP, green fluorescent protein; KV, Kupffer’s vesicle; sox, SRY-box transcription factor; Tg, transgene. (TIF) [file pbio.3000203.s004.tif]

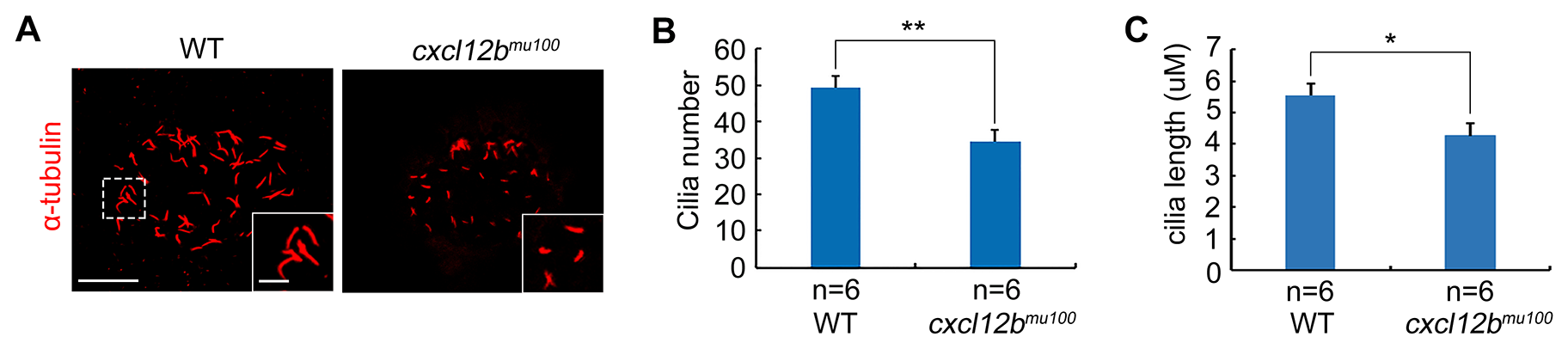

Supplement: S5 Fig — Wild-type embryos and cxcl12bmu100 mutants were harvested at the 10-somite stage for fluorescent immunostaining using anti-α-Tubulin antibody (A). Scale bar, 20 μm. Cilia average number and length were quantified from three independent experiments, and the group values were expressed as the mean ± SD (B and C). Student t test, *P < 0.05, **P < 0.01. Underlying data can be found in S1 Data. KV, Kupffer’s vesicle; α-Tubulin, acetylated tubulin. (TIF) [file pbio.3000203.s005.tif]

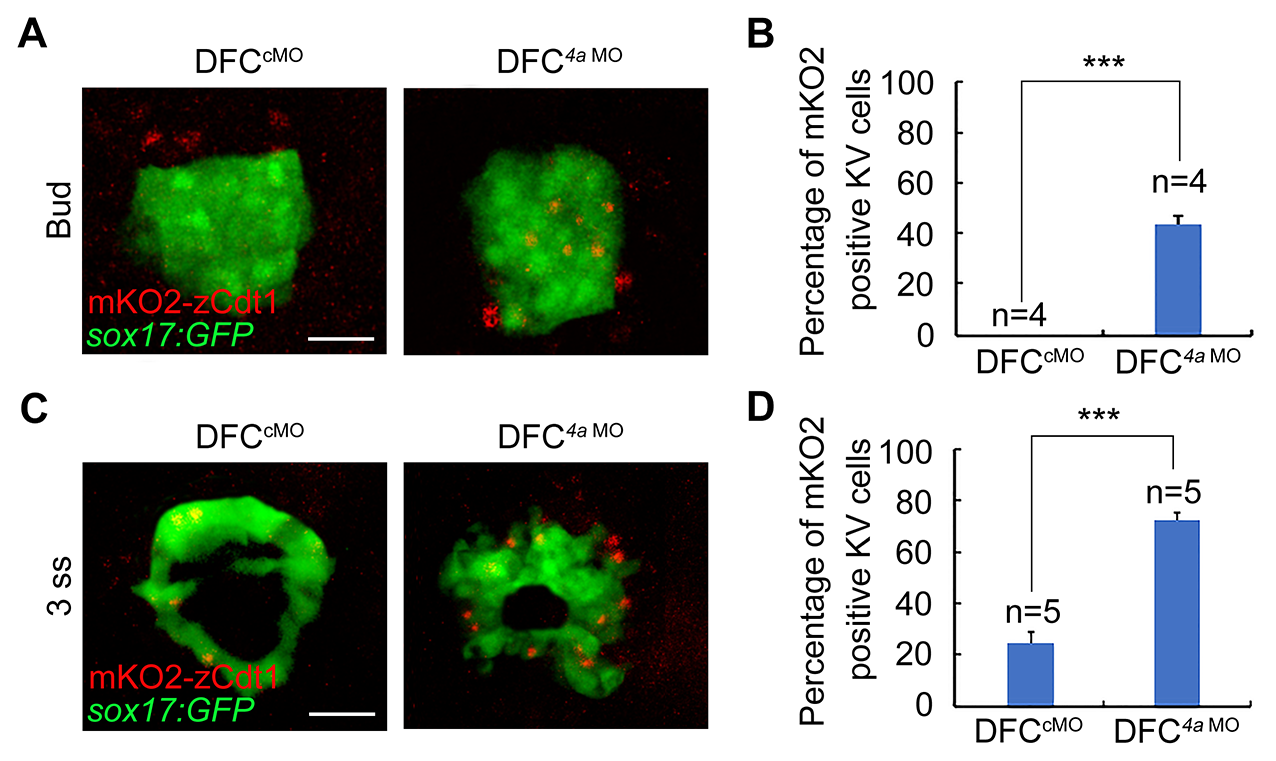

Supplement: S6 Fig — Tg(sox17:GFP;EF1α:mKO2-zCdt1(1/190)) embryos were injected with 8 ng cMO or cxcr4a MO at the 256-cell stage, and then harvested at the indicated developmental stages for in vivo confocal imaging (A and C). Scale bar, 20 μm. The percentage of mKO2-positive KV cells were quantified from three independent experiments (B and D). The significance of differences compared with the control group were analyzed with the Student t test, ***P < 0.001. Underlying data can be found in S1 Data. cMO, control MO; EF1α, eukaryotic translation elongation factor 1α; GFP, green fluorescent protein; KV, Kupffer’s vesicle; mKO2, monomeric Kusabira Orange2; MO, morpholino; sox, SRY-box transcription factor; Tg, transgene; zCdt1, zebrafish chromatin licensing and DNA replication factor 1. (TIF) [file pbio.3000203.s006.tif]

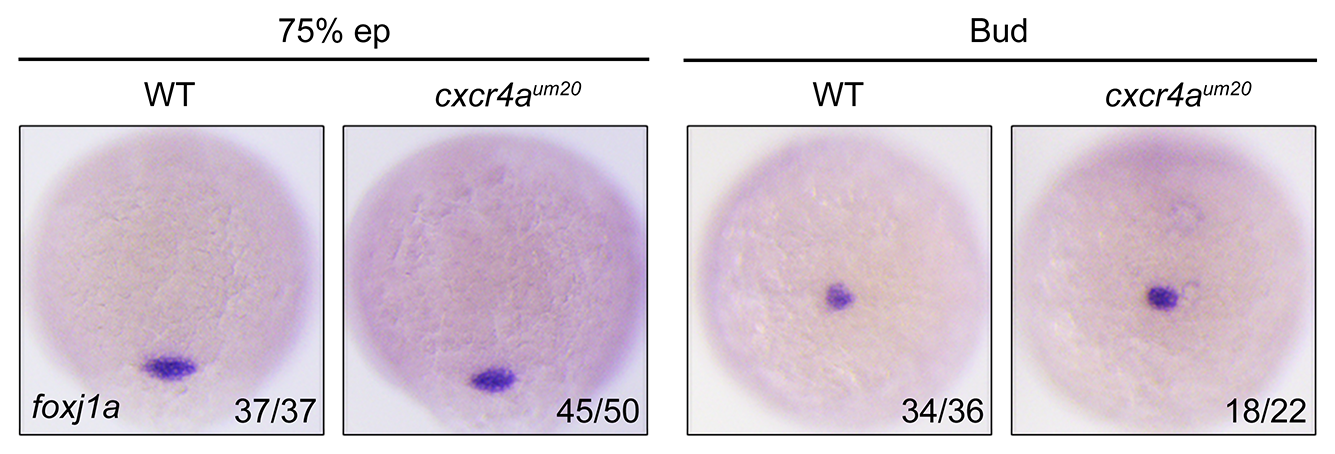

Supplement: S7 Fig — foxj1a expression was examined by in situ hybridization at the 75% epiboly and bud stages in wild-type and cxcr4aum20 mutant embryos. Foxj1a, forkhead box j1a. (TIF) [file pbio.3000203.s007.tif]

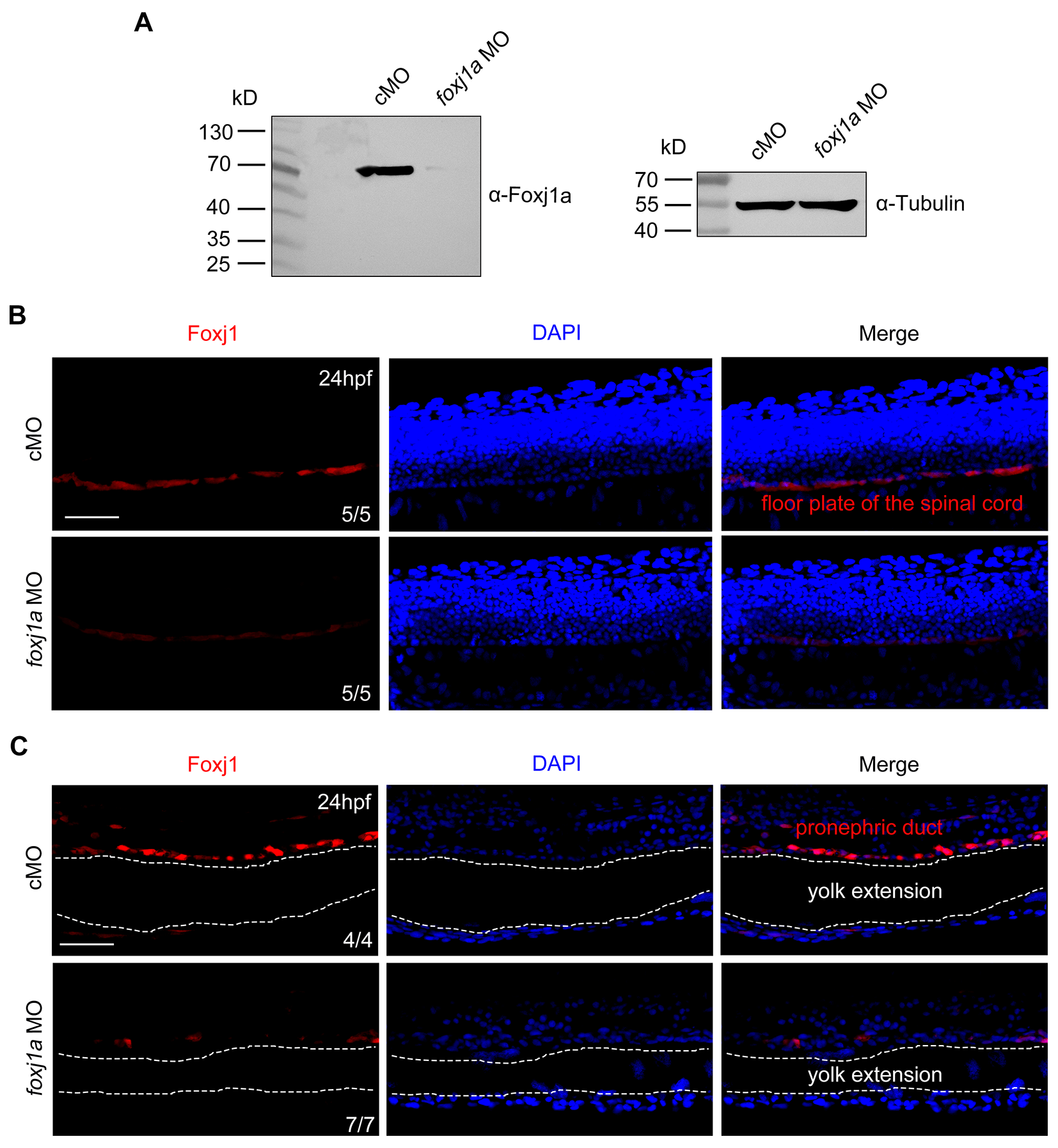

Supplement: S8 Fig — (A) Wild-type embryos were injected with 3 ng cMO or zfoxj1a MO at the one-cell stage and harvested for western blotting at the 75% epiboly stage. (B and C) Detection of zFoxj1a protein in the floor plate of the spinal cord and pronephric duct. cMO- and zfoxj1a MO-injected embryos at 24 hpf were stained with anti-FOXJ1 antibody and DAPI. The floor plate of the spinal cord (B) and pronephric duct (C) were observed after immunostaining. Note that the expression of zFoxj1a protein was significantly decreased in foxj1a morphants. Scale bar, 50 μm. cMO, control MO; Foxj1a, forkhead box j1a; hpf, hours postfertilization; MO, morpholino; zFoxj1a, zebrafish Foxj1a. (TIF) [file pbio.3000203.s008.tif]

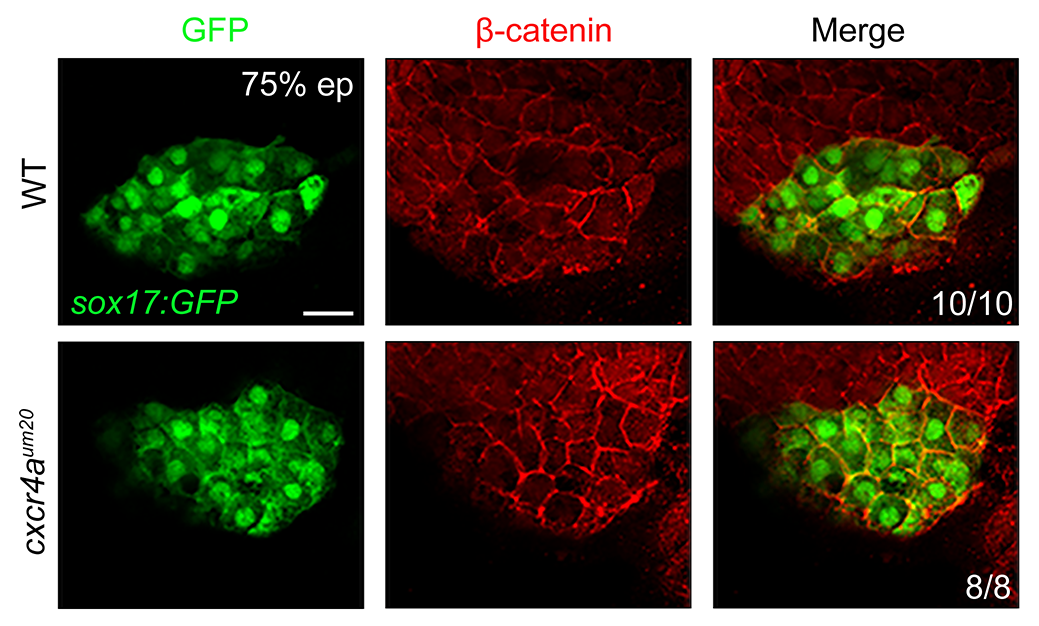

Supplement: S9 Fig — Wild-type and cxcr4aum20 mutants were harvested at the 75% epiboly stage for immunofluorescence assays using the indicated antibodies. Scale bar, 20 μm. DFC, dorsal forerunner cell. (TIF) [file pbio.3000203.s009.tif]

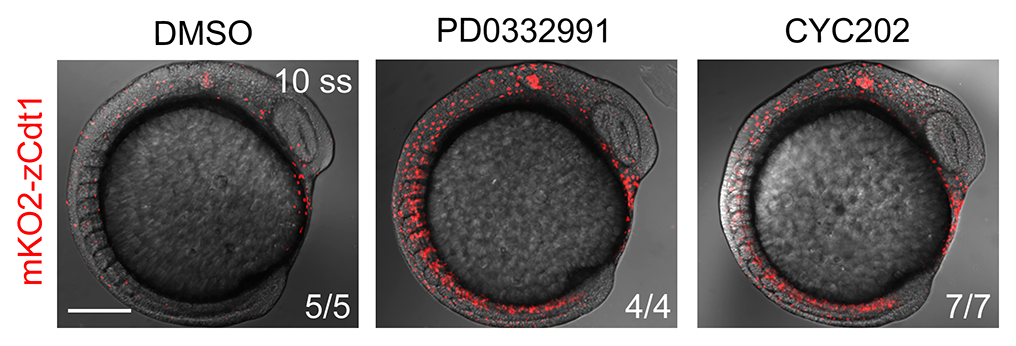

Supplement: S10 Fig — Tg(mKO2-zCdt1(1/190)) embryos were treated with 0.5 μM PD0332991 or 0.2 μM CYC202 from the shield stage to the 10-somite stage. Then, these embryos were harvested for in vivo confocal imaging. Note that both PD0332991 and CYC202 treatments induced a remarkable increase of the number of mKO2-zCdt1–positive cells. Scale bar, 200 μm. CDK, cyclin-dependent kinase; EF1α, eukaryotic translation elongation factor 1α; mKO2, monomeric Kusabira Orange2; Tg, transgene; zCdt1, zebrafish chromatin licensing and DNA replication factor 1. (TIF) [file pbio.3000203.s010.tif]

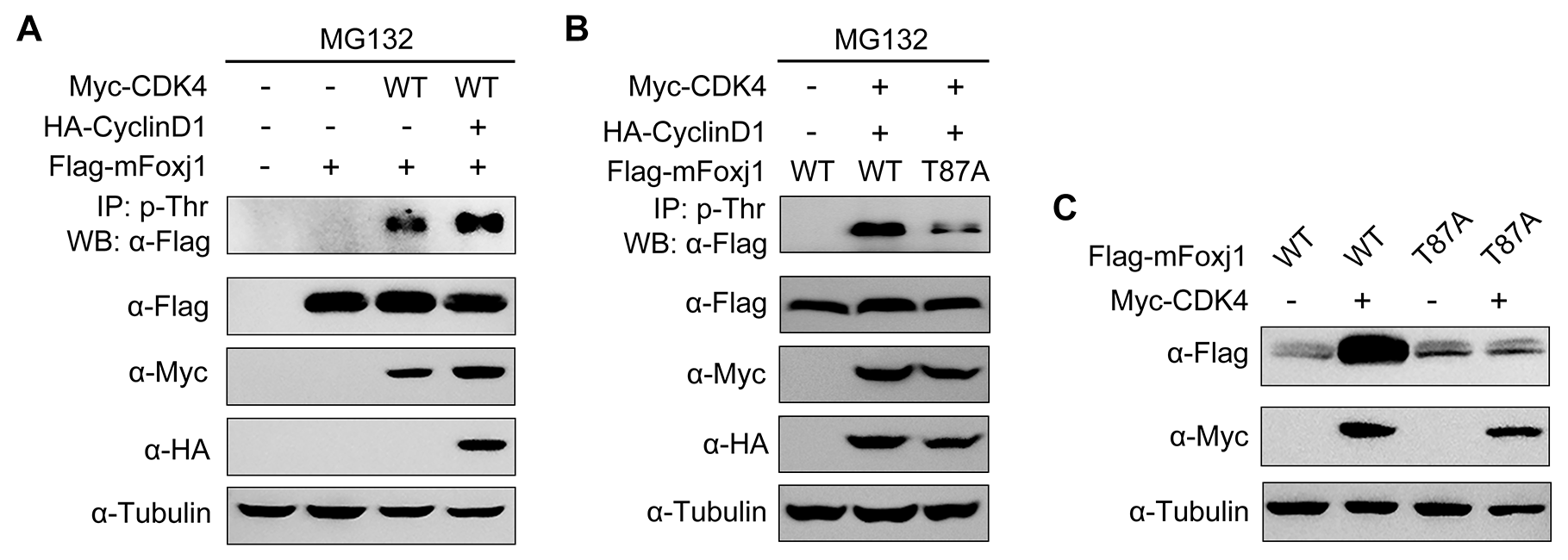

Supplement: S11 Fig — (A–B) HEK293T cells were transfected with the indicated plasmids and then harvested for immunoprecipitation with a phospho-threonine–proline antibody. Phosphorylation of mFoxj1 (A) and its T87A mutant (B) was detected by western blot. Note that the CDK4-mediated phosphorylation of mFoxj1 was clearly decreased in the T87A mutant. (C) Western blots of total lysates from HEK293T cells transfected with the indicated plasmids. Note that CDK4 overexpression could stabilizes wild-type mFoxj1 but not the T87A mutant. CDK, cyclin-dependent kinase; HEK, human embryonic kidney; Foxj1, forkhead box j1a; mFoxj1, mouse Foxj1. (TIF) [file pbio.3000203.s011.tif]

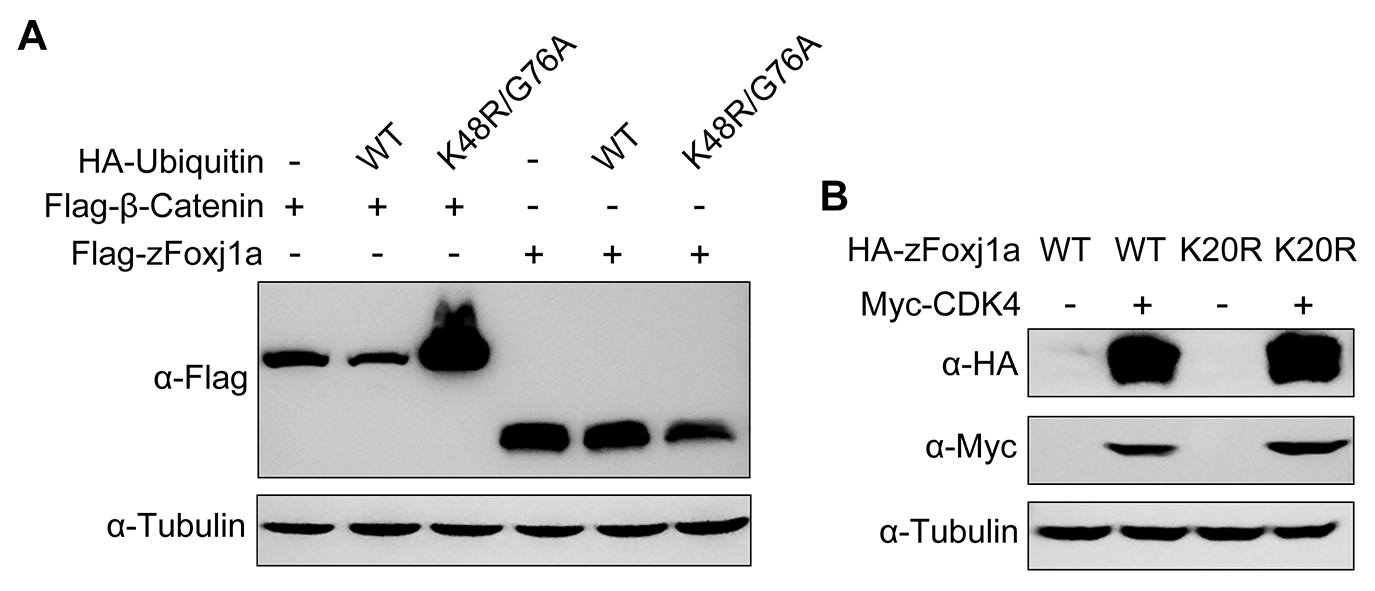

Supplement: S12 Fig — (A) Overexpression of Ub K48R/G76A was unable to stabilize zFoxj1a. Flag-tagged β-catenin and zFoxj1a were coexpressed with wild-type Ub or Ub K48R/G76A, a dominant negative inhibitor of chain formation and degradation. Cell extracts were immunoblotted with the indicated antibodies. (B) CDK4 overexpression similarly promoted the expression of wild-type zFoxj1a and its lysineless mutant K20R. CDK, cyclin-dependent kinase; Foxj1a, forkhead box j1a; Ub, ubiquitin; zFoxj1a, zebrafish Foxj1a. (TIF) [file pbio.3000203.s012.tif]

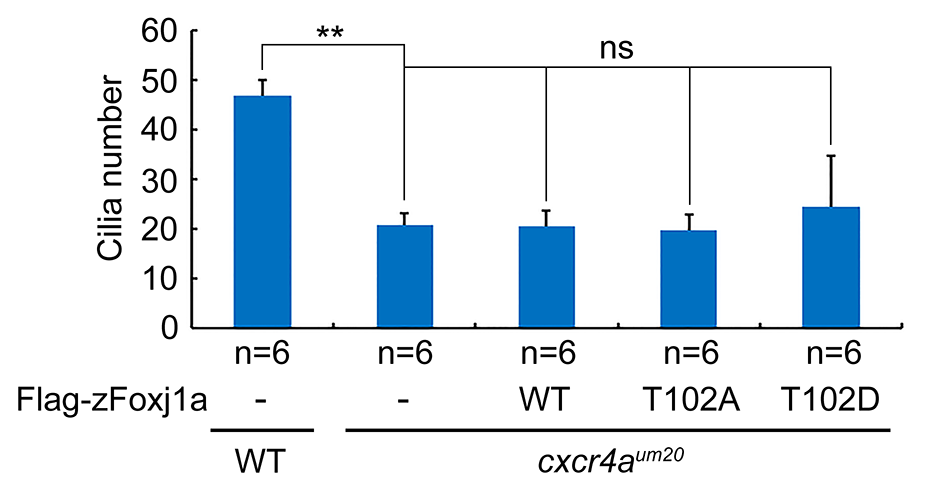

Supplement: S13 Fig — cxcr4a-deficient embryos were injected with 200 pg of wild-type zfoxj1a or zfoxj1a-T102A or zfoxj1a-T102D mRNA at the 256-cell stage. The resulting embryos were harvested at the 10-somite stage for immunostaining using an antibody against α-Tubulin. Cilia number was quantitatively analyzed using ImageJ software. Student t test, **P < 0.01. Underlying data can be found in S1 Data. DFC, dorsal forerunner cell; Foxj1a, forkhead box j1a; ns, no significant difference; T102, threonine 102; zFoxj1a, zebrafish Foxj1a; α-Tubulin, acetylated tubulin. (TIF) [file pbio.3000203.s013.tif]

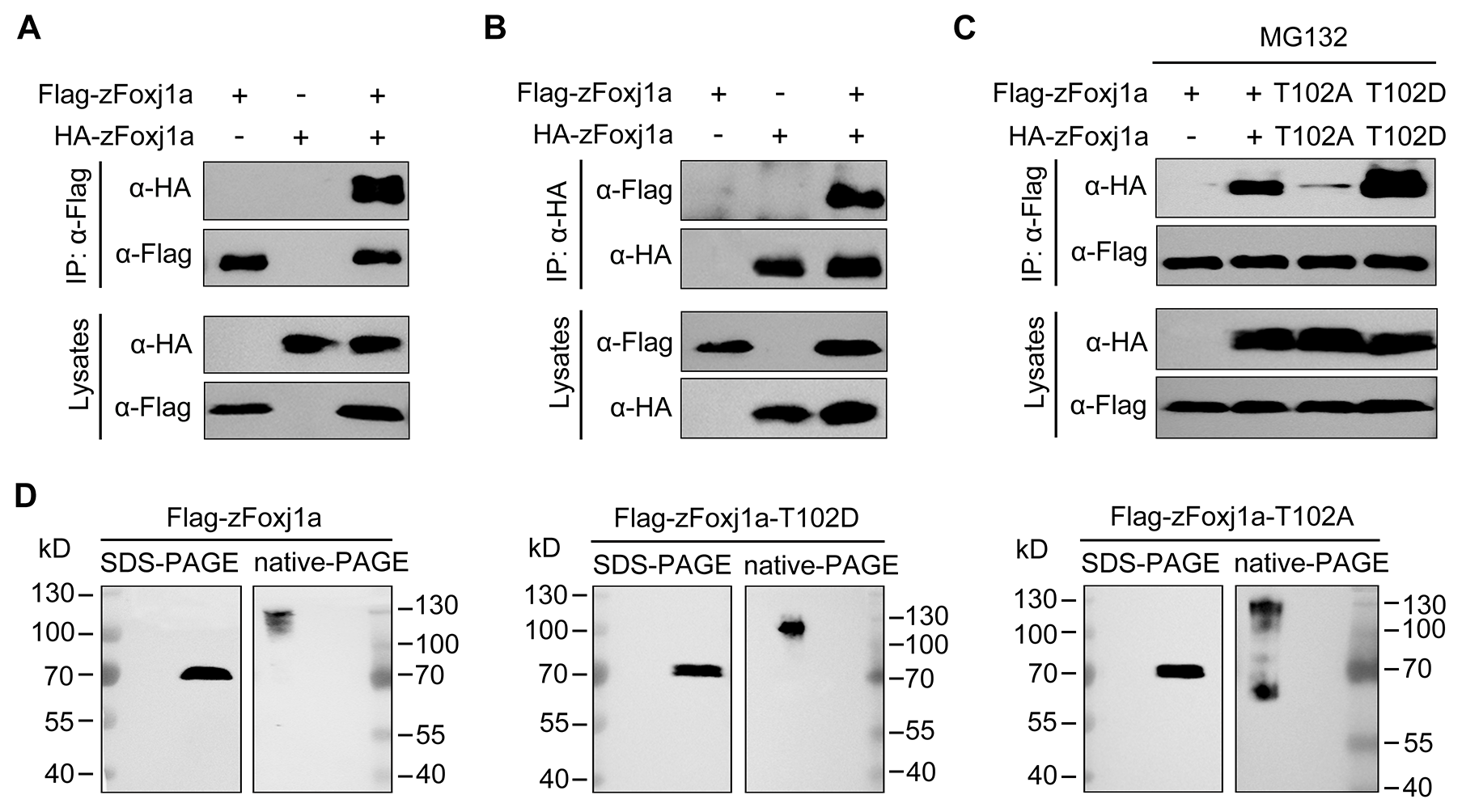

Supplement: S14 Fig — (A–C) In vivo self-association of zFoxj1a. HEK293T cells were transfected with indicated plasmids encoding differently tagged wild-type zFoxj1a or its mutants. Lysates were immunoprecipitated with anti-Flag or anti-HA antibodies and then immunoblotted with indicated antibodies. In panel C, HEK293T cells were treated with MG132 for 5 hours prior to harvest for immunoprecipitation. (D) zFoxj1a forms homodimers. HEK293T cells were transfected with indicated plasmids encoding Flag-tagged wild-type zFoxj1a or its mutants. Lysates were then separated on SDS- and native PAGEs, respectively. Foxj1a, forkhead box j1a; HA, hemagglutinin; HEK, human embryonic kidney; T102, threonine 102; zFoxj1a, zebrafish Foxj1a. (TIF) [file pbio.3000203.s014.tif]
