# Supplementary material for: Chemokine signaling links cell-cycle progression and cilia formation for left–right symmetry breaking
Source: PLoS Biol. 2019 Aug 20;17(8):e3000203. doi: 10.1371/journal.pbio.3000203 (PMC6716676; doi:10.1371/journal.pbio.3000203)

Uncropped blots for Figure 3J

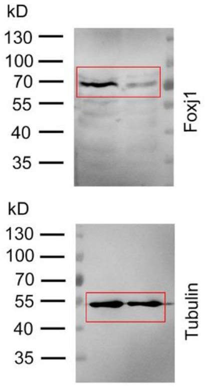

Uncropped blots for Figure 5D

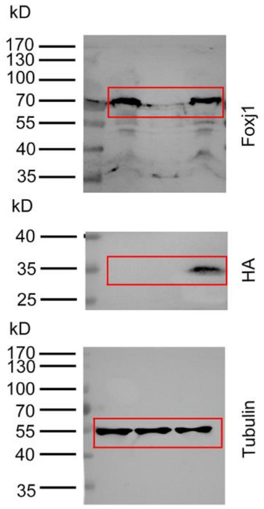

Uncropped blots for Figure 5E

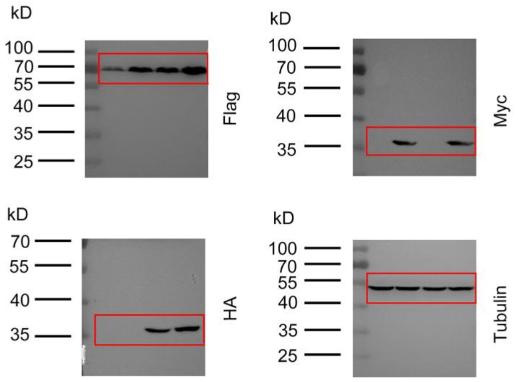

Uncropped blots for Figure 5F

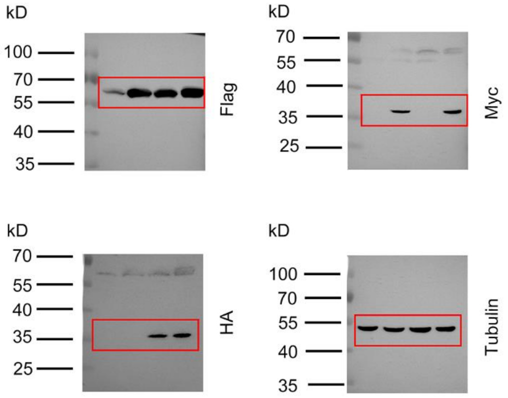

Uncropped blots for Figure 5G

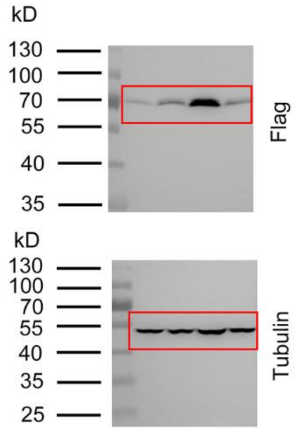

Uncropped blots for Figure 5H

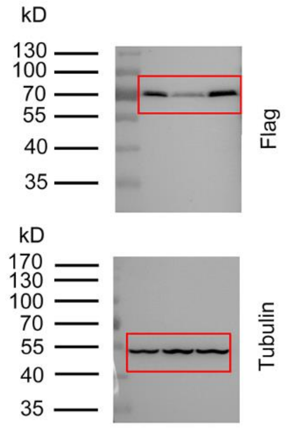

Uncropped blots for Figure 5I

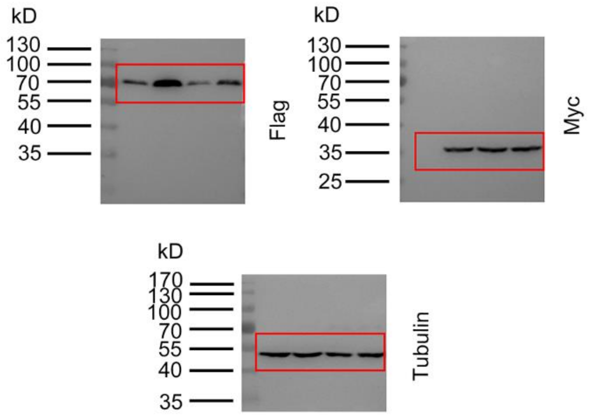

Uncropped blots for Figure 6B

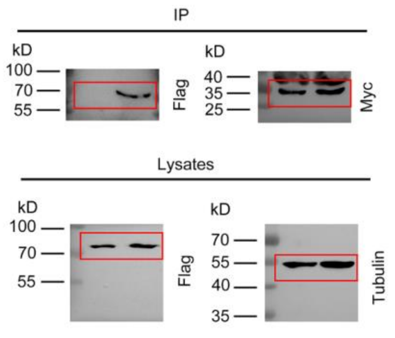

Uncropped blots for Figure 6F

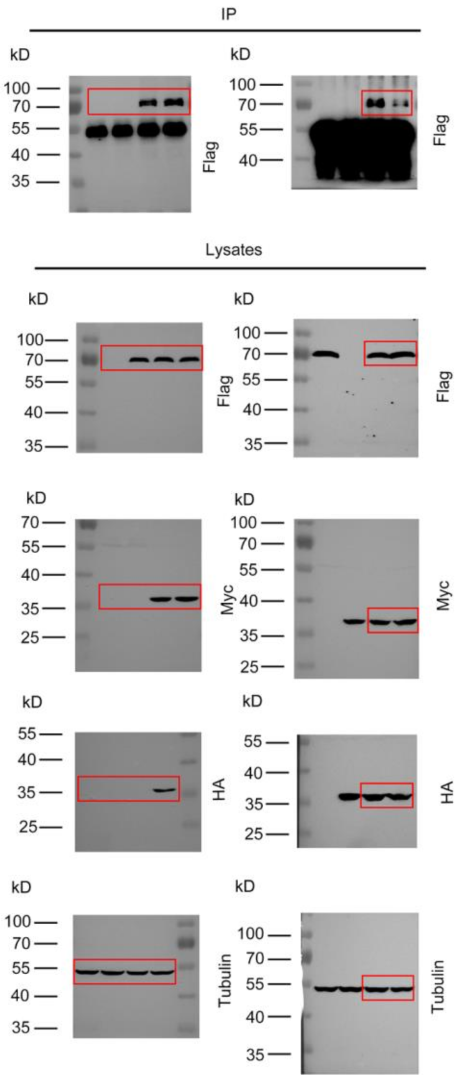

Uncropped blots for Figure 6C

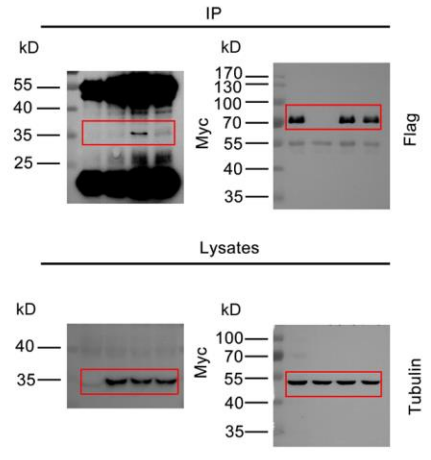

Uncropped blots for Figure 6D

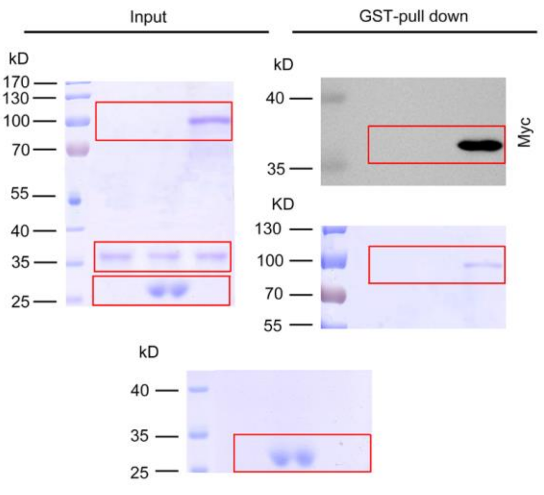

Uncropped blots for Figure 6G

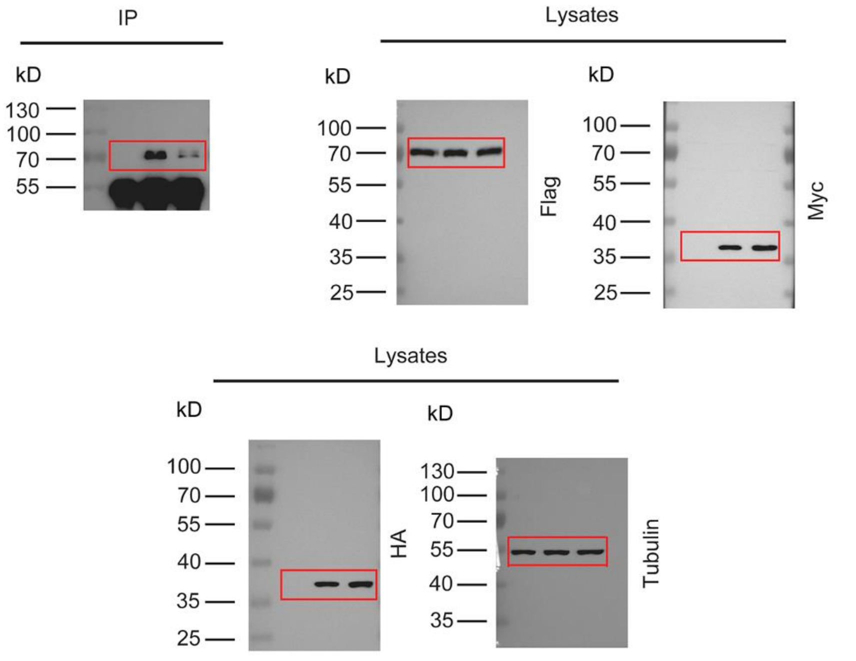

Uncropped blots for Figure 6H

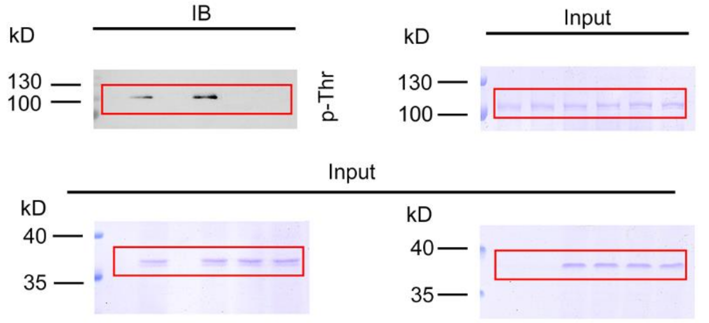

Uncropped blots for Figure 6I

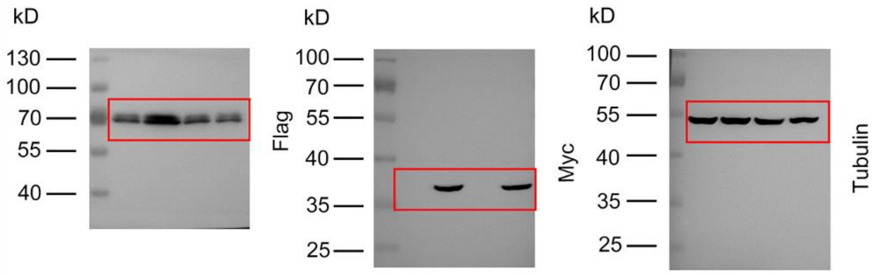

Uncropped blots for Figure 7A

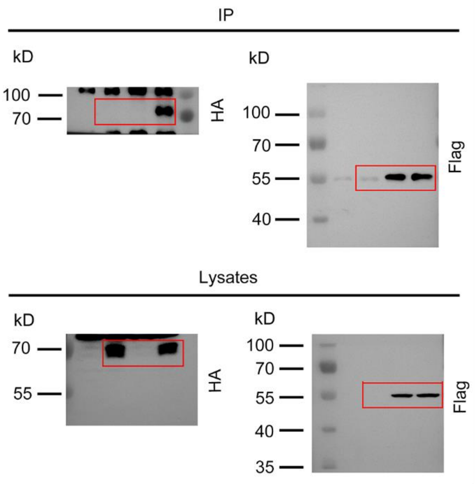

Uncropped blots for Figure 7B

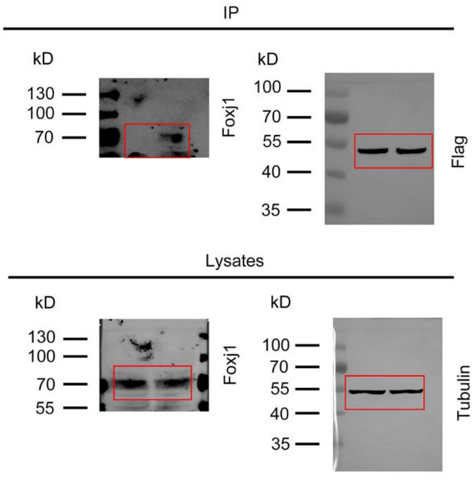

Uncropped blots for Figure 7C

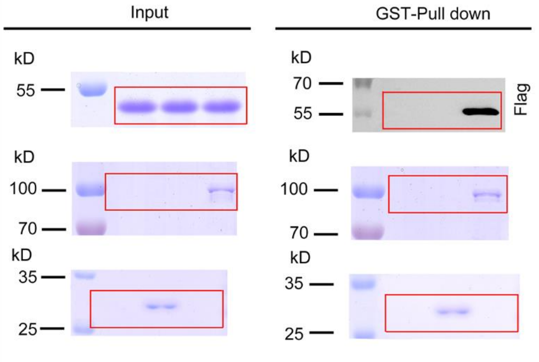

Uncropped blots for Figure 7D

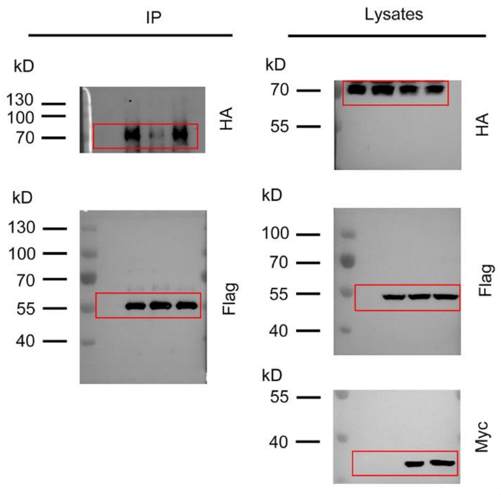

Uncropped blots for Figure 7E

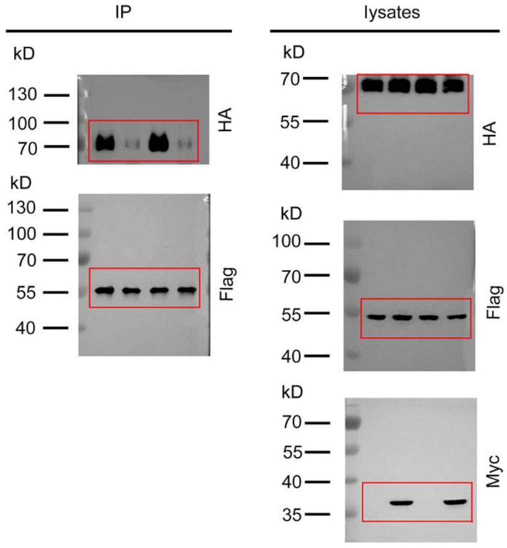

Uncropped blots for Figure 7F

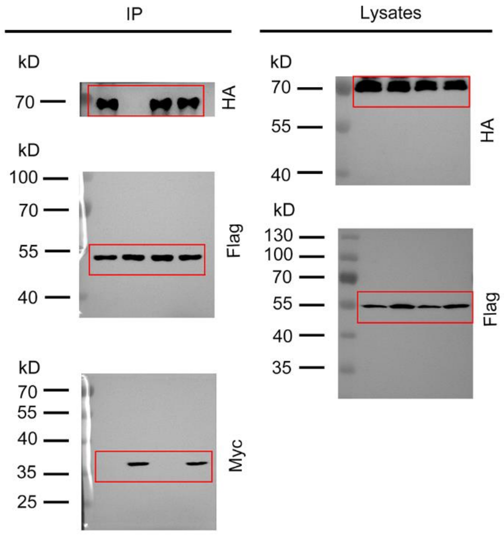

Uncropped blots for Figure 7G

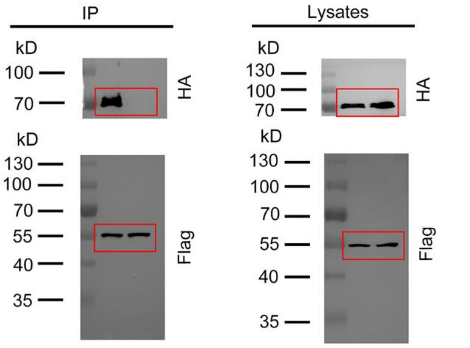

Uncropped blots for Figure 7H

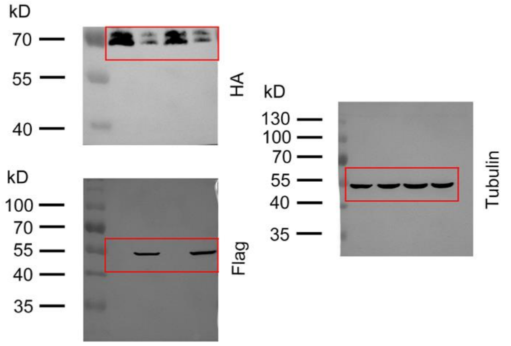

Uncropped blots for Figure 7I

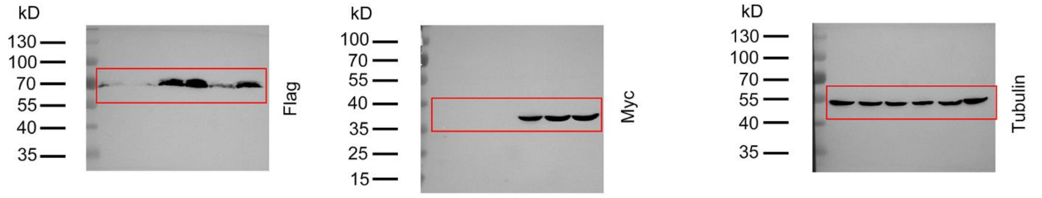

Uncropped blots for Figure S6A

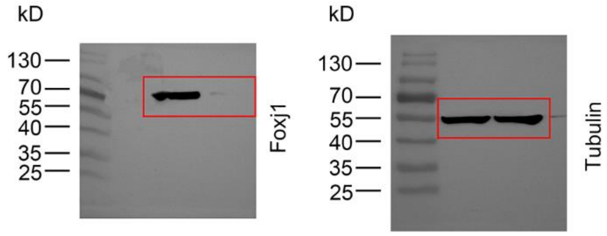

Uncropped blots for Figure S9A

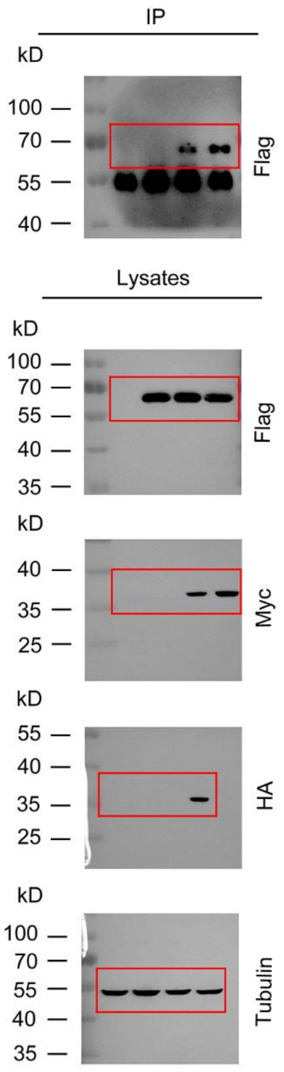

Uncropped blots for Figure S9B

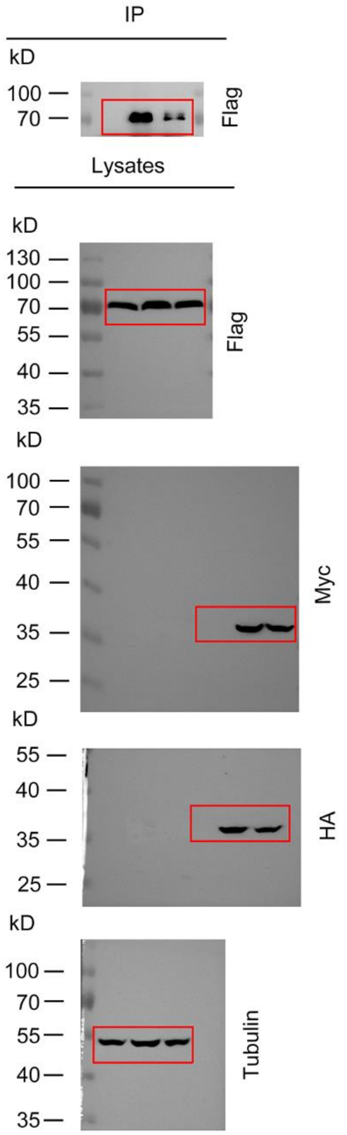

Uncropped blots for Figure S9C

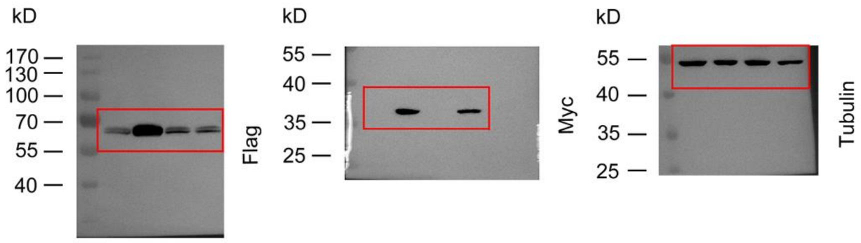

Uncropped blots for Figure S10B

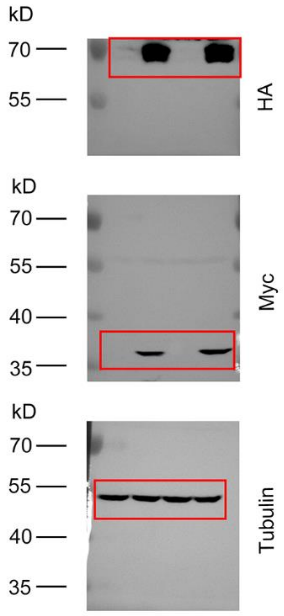

Uncropped blots for Figure S10A

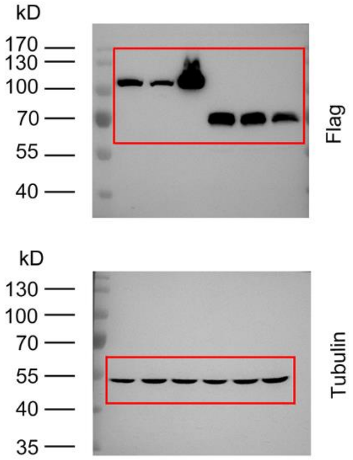

Uncropped blots for Figure S12A

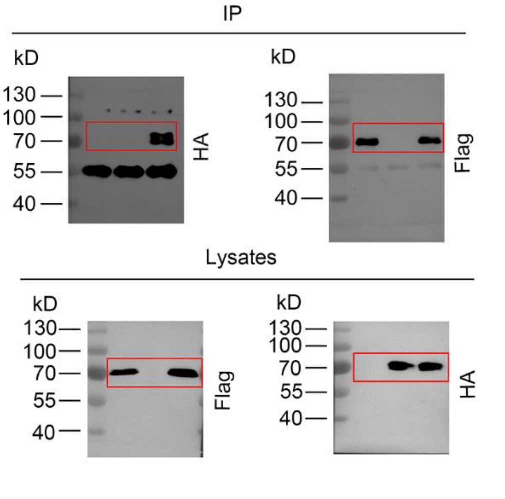

Uncropped blots for Figure S12B

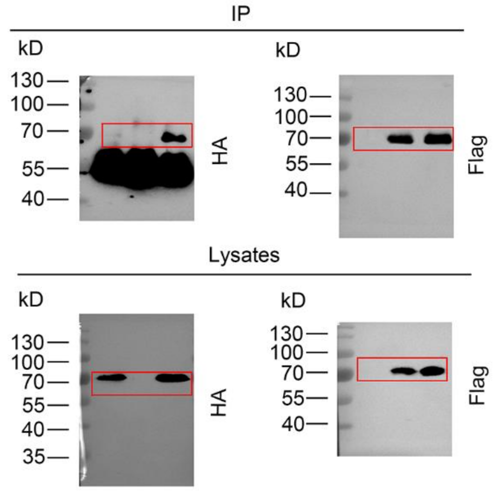

Uncropped blots for Figure S12C

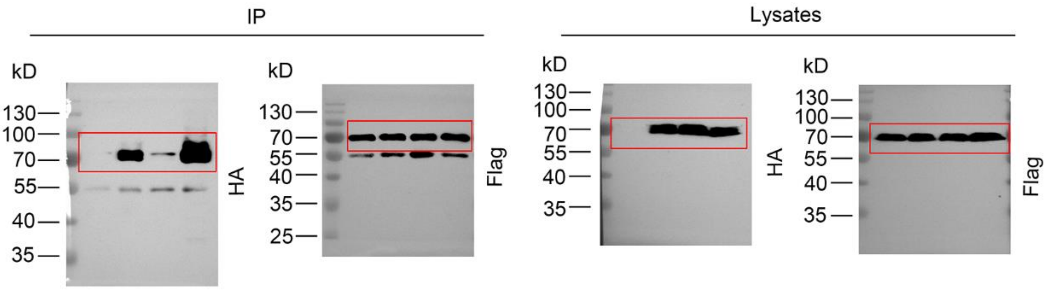

Uncropped blots for Figure S12D

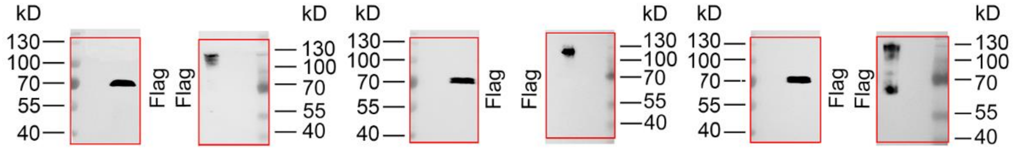

Supplement: S1 Raw Images — (PDF) [file pbio.3000203.s015.pdf]
